# Immuno-pharmacokinetics of Meglumine Antimoniate in Patients With Cutaneous Leishmaniasis Caused by *Leishmania* (*Viannia*)

**DOI:** 10.1093/cid/ciaa1206

**Published:** 2020-08-20

**Authors:** María Adelaida Gómez, Adriana Navas, Miguel Dario Prieto, Lina Giraldo-Parra, Alexandra Cossio, Neal Alexander, Nancy Gore Saravia

**Affiliations:** 1 Centro Internacional de Entrenamiento e Investigaciones Médicas-CIDEIM, Cali, Colombia; 2 Universidad Icesi, Cali, Colombia

**Keywords:** pharmacokinetics, pharmacodynamics, *Leishmania*, hysteresis, antimonials

## Abstract

**Background:**

Control of cutaneous leishmaniasis (CL) relies on chemotherapy, yet gaps in our understanding of the determinants of therapeutic outcome impede optimization of antileishmanial drug regimens. Pharmacodynamic (PD) parameters of antimicrobials are based on the relationship between drug concentrations/exposure and microbial kill. However, viable *Leishmania* persist in a high proportion of individuals despite clinical resolution, indicating that determinants other than parasite clearance are involved in drug efficacy.

**Methods:**

In this study, the profiles of expression of neutrophils, monocytes, T_h_1 and T_h_17 gene signatures were characterized in peripheral blood mononuclear cells (PBMCs) during treatment with meglumine antimoniate (MA) and clinical cure of human CL caused by *Leishmania* (*Viannia*). We explored relationships of immune gene expression with plasma and intracellular antimony (Sb) concentrations.

**Results:**

Our findings show a rapid and orchestrated modulation of gene expression networks upon exposure to MA. We report nonlinear pharmacokinetic/pharmacodynamic (PK/PD) relationships of Sb and gene expression dynamics in PBMCs , concurring with a time lag in the detection of intracellular drug concentrations and with PK evidence of intracellular Sb accumulation.

**Conclusions:**

Our results quantitatively portray the immune dynamics of therapeutic healing, and provide the knowledge base for optimization of antimonial drug treatments, guiding the selection and/or design of targeted drug delivery systems and strategies for targeted immunomodulation.

## Teaser

We describe the nonlinear relationship between meglumine antimoniate pharmacokinetics and immune gene expression profiles during treatment of patients with cutaneous leishmaniasis and reveal immune gene signatures as pharmacodynamic end points of antileishmanial drugs.

Optimization of antileishmanial drug regimens to enhance efficacy and reduce toxicity is hampered by poor understanding of the bases of the therapeutic response. As occurs in other infections, treatment failure and response occur during infections with drug-susceptible as well as drug-resistant *Leishmania* [1–3], suggesting that the *s*usceptibility parameters may be inadequate on their own and that other factors may be critical to achieve clinical cure. The efficacy of antileishmanial drugs is dependent on immunocompetence [4–6], yet treatment failure occurs in approximately 30% of apparently immunocompetent individuals [7–9]. Furthermore, reduced drug concentrations or exposures have been shown to contribute to treatment failure with antimonials and miltefosine [10, 11]. However, lack of information on the pharmacokinetic/pharmacodynamic (PK/PD) parameters associated with efficacy or the concentration thresholds that predict outcome limit optimization of drug regimens. PK studies of antileishmanials have been based on plasma drug concentrations under the assumption that these reflect intracellular concentrations. However, with the exception of miltefosine [12, 13], the relationship between plasma and intracellular antileishmanial PK is unknown.

The following two features of the pathobiology of cutaneous leishmaniasis (CL) critically influence the efficacy of antileishmanials: the intracellular habitat of *Leishmania* and the elicitation of immunopathologic mechanisms by and during infection [14]. The ability of *Leishmania* to modulate innate and adaptive immune responses, the persistent nature of infection, and the involvement of deregulated inflammation in development of pathology and clinical outcomes of CL caused by *Leishmania* (*Viannia*), suggest that alteration of these processes is central to disease resolution. However, PD targets of antileishmanial drugs, as of other antimicrobials, are based on the parameter of microbial burden, despite persistence of *Leishmania* in approximately 40% of clinically cured cases [15–17]. Thus, the intracellular PK/PD of drugs and the effects of drug exposure on immune responses could be key in optimizing available regimens and in developing novel interventions.

## METHODS

### Patients

Fourteen adult patients (aged 18–52 years) with parasitological diagnosis of CL and clinical manifestations of disease <6 months of evolution participated in this study (Table 1). Exclusion criteria included pregnancy, mucosal leishmaniasis, use of any antileishmanial drug in the 3 months prior to enrollment, a human immunodeficiency virus–positive test, and presence of clinical and laboratory contraindications for antimonial treatment.

**Table 1.** Clinical and Demographic Characteristics of Study Participants

Characteristic	Total
Number of participants	14
Sex, n (%)	
Male	13 (93)
Female	1 (7)
Age, mean (SD), y	31.4 (±11)
Weight, mean (SD), kg	76.2(±12.6)
Time of disease evolution, mean (SD), mo	2.5 (±0.7)
Number of lesions per patient, median (range)	1 (1–4)
Adherence to treatment (ampules prescribed vs ampules administered), median (range)	100 (70–100)
Intensity of Adverse Drug Reactions (ADR), n (%)	
Mild	70 (83.3)
Moderate	14 (16.7)
Severe	0 (0)

Abbreviation: SD, standard deviation.

### Procedures and Samples

For CL patients, baseline evaluations were performed that included blood cell counts and assessment of cardiac, pancreatic, liver, and renal function. Patients were treated with parenteral meglumine antimoniate (MA; 20 mg antimony [Sb]/kg every 24 hours for 20 days) [18]. Clinical and laboratory follow-up included visits at the first day of treatment, at day 10 during treatment and day 20 (end of treatment [EoT]), and at weeks 8 and 13 (±2 weeks) after initiation of treatment, the latter time at which clinical outcome was determined. Cure was defined as complete reepithelialization, absence of inflammatory signs for all CL lesions, and absence of new leishmaniasis lesions [19].

Blood specimens were obtained prior to initiation of treatment and 1 hour postdose on days 1, 10, and 20 and at weeks 8 and 13 after initiation of treatment. Patients remained at Centro Internacional de Entrenamiento e Investigaciones Médicas (CIDEIM) outpatient clinics in Cali, Colombia, on day 20 when the PK sampling was conducted with sequential samples obtained at 0, 0.5, 1.0, 1.5, 2, 3, 5, 8, 12, and 24 hours after the final dose (see Supplementary Figure 1). Plasma and peripheral blood mononuclear cells (PBMCs) were isolated from these samples.

### Antimony Measurements and PK Analyses

Total Sb concentrations were measured in plasma and PBMC pellet digests using inductively coupled plasma mass spectrometry (ICP-MS) on an Agilent 7700x instrument. This method measures total Sb content (Sb^III^ and Sb^V^, indistinctively). Details of the ICP-MS are provided in the Supplementary Material. The PK parameters of Sb in plasma and PBMCs were determined by noncompartmental analyses using the PKNCA package for R software (version 3.6.1) [20, 21].

### Real-time Quantitative Polymerase Chain Reaction

Custom made polymerase chain reaction (PCR) arrays (Qiagen, CLAH23658D) were constructed to analyze the expression of 27 inflammatory mediators (Supplementary Table 1), selected based on systematic profiling of skin lesion biopsies and PBMCs from a previously characterized CL patient cohort [22]. Gene expression was measured using real-time quantitative PCR. Data were normalized using 2 housekeeping genes: glyceraldehyde-3-phosphate dehydrogenase and ribosomal protein large P0. Fold-change gene expression was calculated using the ΔΔCt method. Additional information on RNA extraction is available in the Supplementary Methods.

### Statistical Analyses

Differences in mean of quantitative data were estimated using Mann-Whitney and *t* tests. Differences in proportions were determined using χ ^2^ or Fisher exact tests. Gene expression data (2^-Δct^) were log-transformed and used as input for network analyses using Graphia Professional Software (Kajeka Ltd, United Kingdom) with a set parameter for Spearman correlation cutoff |ρ| > 0.75 [23]. The Markov clustering algorithm [24] embedded within Graphia was used for unsupervised clustering of gene expression datasets. Relationships between plasma and intracellular drug concentrations and gene expression profiles were analyzed in concentration-time curves as well as time-dependent concentration-effect curves. Data analysis was done in R version 3.6.1 and Graph Pad Prism software V. 6.07.

### Ethics Statement

This study was approved and monitored by the institutional review board for ethical conduct of research involving human subjects of CIDEIM in accordance with Colombian and international guidelines. All individuals voluntarily participated in the study and provided written informed consent before inclusion.

## RESULTS

### PBMCs Accumulate Antimony During In Vivo Treatment With Meglumine Antimoniate

**Figure 1.**
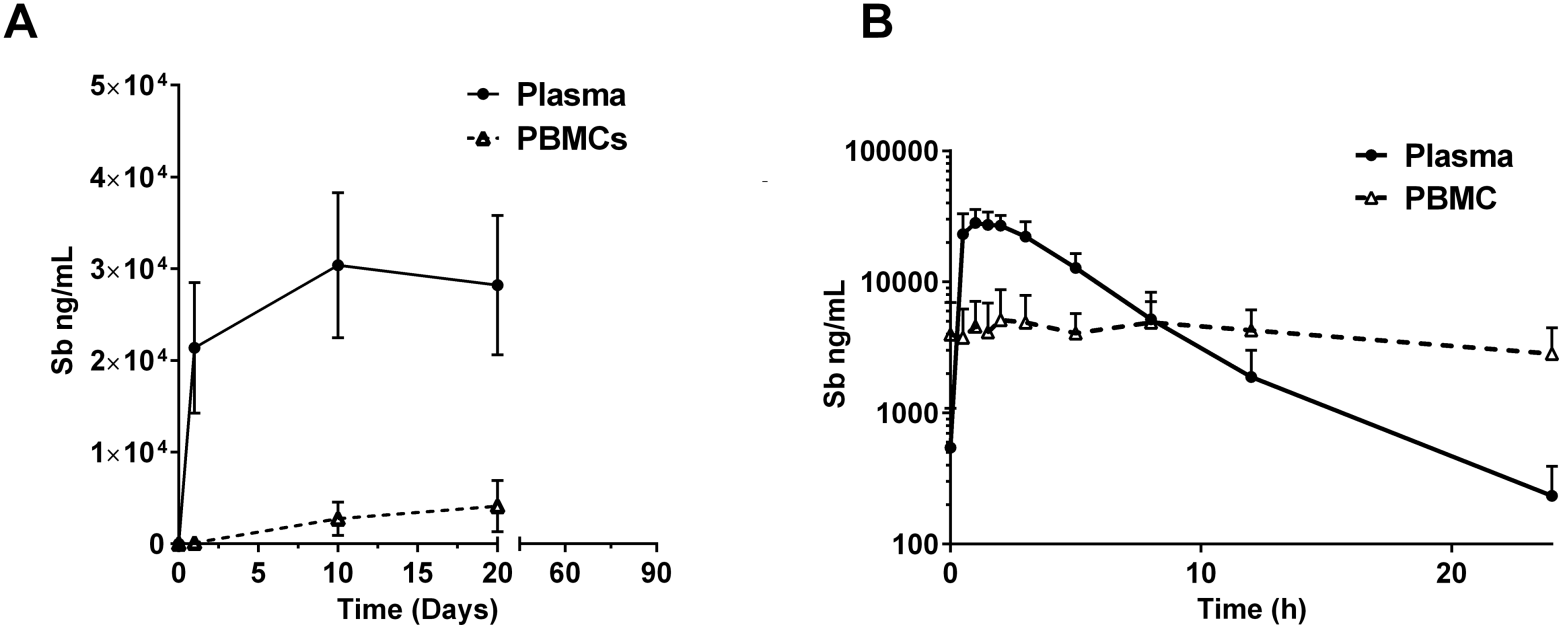
Concentration-time curves of antimony in plasma and PBMC samples. Antimony concentrations measured in plasma and PBMCs 1 hour after dosing over the course of treatment (*A*) and hourly during the last day of treatment (*B*). Samples were obtained from 10 adult cutaneous leishmaniasis patients throughout the course of treatment and up to 3 months of follow-up. Data are shown as mean values ± standard deviation. Abbreviation: PBMC, peripheral blood mononuclear cell.

Fourteen CL patients undergoing antileishmanial treatment with MA participated in this study. Clinical and demographic characteristics of participants evidenced homogeneity of the study group (Table 1). All patients were cured and reported mild to moderate adverse drug reactions (ADR). Total Sb concentrations in plasma ([Sb_p_]) were detected throughout treatment in samples collected 1 hour after dosing (Supplementary Figure 1). Samples collected on days 60 and 90 after initiation of treatment were all under the lower limit of quantitation (LLOQ, 25 ng/mL) of the ICP-MS method (Figure 1A). Intracellular Sb concentrations ([Sb_i_]) were also measured within PBMCs. [Sb_i_] measured 1 hour after dose on the first day of treatment was under the LLOQ but measurable thereafter in samples collected 1 hour after dosing on days 10 and 20 (EoT). [Sb_i_] at days 60 and 90 were under the LLOQ (Figure 1A).

Concentration-time curves and PK parameters were derived from [Sb_p_] measured in samples collected over a 24-hour period at EoT (Figure 1B). Plasma and intracellular PK parameters from noncompartmental analyses are shown in Table 2. The maximum plasma concentration was rapidly achieved (C_max_ = 30 190 μg/L ± 2414 μg/L; T_max_ = 1.4 hours). Intracellular time to reach Cmax (T_max_) was 3.4 hours and was 5 times lower than plasma C_max_ (intracellular C_max_ = 6625 μg/L ± 1186 μg/L). The trough [Sb_i_] (C_24i_) was 2739 μg/L (± 497 μg/L), which was 10 times higher than plasma C_24_ (234 μg/L ± 50.3 μg/L). The slower time-dependent decrease in [Sb_i_] compared with [Sb_p_] provides evidence of intracellular drug accumulation.

**Table 2.** Plasma and Intracellular Noncompartmental Pharmacokinetic Analyses

Parameter^a^	Plasma	Intracellular: Peripheral Blood Mononuclear Cells
Area under the time-concentration curve in plasma from 0 to 24 hours after the dose, µg-h/L	152 248 (13 773)	89 078 (14 261)
Drug clearance, adjusted for bioavailability and divided by body weight, L/h-kg	0.139 (0.0109)	Not applicable
Maximum plasma concentration, µg/L	30 190 (2414)	6625 (1186)
Time to reach Cmax (T_max_), h	1.40 (0.19)	3.40 (0.85)
Trough concentration at the end of the usual 24-hour dosing interval (observed value), µg/L	234 (50.3)	2739 (497)
Half-life, h	3.42 (0.19)	31.1 (4.1)^b^
Apparent volume of distribution, divided by body weight, L/kg	0.680 (0.0657)	Not applicable

^a^Parameters are given as mean ± standard error.

^b^Three individuals were missing values because they had fewer than the 3 post-T_max_ values, which are needed to calculate the half-life (on the software’s default settings). Hence, these individuals also have missing drug clearance and apparent volume of distribution because these parameters depend on T_max_ (via area under the time-concentration curve).

The antileishmanial effect of drugs is typically measured in monocytes/macrophages as these are the preferential host cells for *Leishmania*. We asked whether the lower [Sb_i_] could be an artifact of specific accumulation within monocytes. PBMCs from healthy donors were obtained, and monocytes were isolated using CD14+ magnetic bead sorting. Ten million PBMCs and 10 million monocytes were incubated with MA for 1 hour at plasma C_max_ (32 000 μg-Sb/L). Similar [Sb_i_] were found in PBMCs and in isolated monocytes, indicating that Sb is incorporated within monocytes as well as other mononuclear white blood cells.

### Functionally Related Immune Genes Are Coregulated During In Vivo Exposure to Antimonials

We recently reported the expression profile of immune-related genes in lesion biopsies of CL patients undergoing antimonial treatment [22]. This, together with a panel of differentially expressed genes in human macrophages associated with cutaneous pathology caused by *Leishmania (V.) panamensis* [25], informed a gene signature consisting of 27 immune-related genes related to CL pathology and the therapeutic response (Supplementary Table 1).

**Figure 2.**
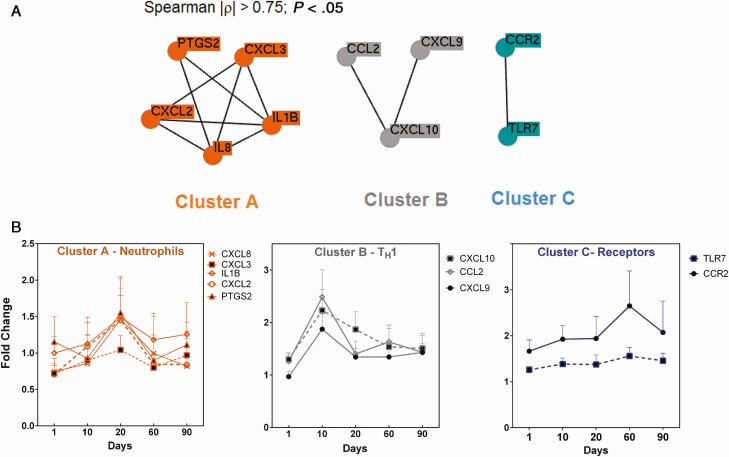
Gene expression networks of peripheral blood mononuclear cells (PBMCs) during cutaneous leishmaniasis (CL) treatment and clinical cure. *A*, Network representation of coregulated or coexpressed genes in PBMCs of CL patients (n = 10). Represented are genes with significant correlated expression (2^-ΔCt^) over the course of meglumine antimoniate treatment (days 1, 10, and 20) and during treatment follow-up (days 60 and 90). *B*, Expression profiles of genes within gene clusters. Data are presented as mean values ± standard deviation of the fold-change difference of each gene at each time point over the gene expression of samples collected pretreatment for each patient.

Expression of this gene signature was analyzed in PBMCs, and a 3-cluster network was defined based on the expression data collected from samples obtained 1 hour after dose on days 1, 10, and 20, as well as samples collected throughout the follow-up period (Figure 2A). One cluster (cluster A) contained 5 genes: PTGS2, IL1β, CXCL3, CXCL2, and CXCL8 (IL8), predominantly reflecting activation and recruitment of neutrophils (Figure 2A). Clusters B and C contained 3 and 2 genes, respectively; cluster B included CXCL9, CXCL10, and CCL2, reflecting a T_H_1 environment; and cluster C, a membrane receptor cluster, contained TLR7 and CCR2.

**Figure 3.**
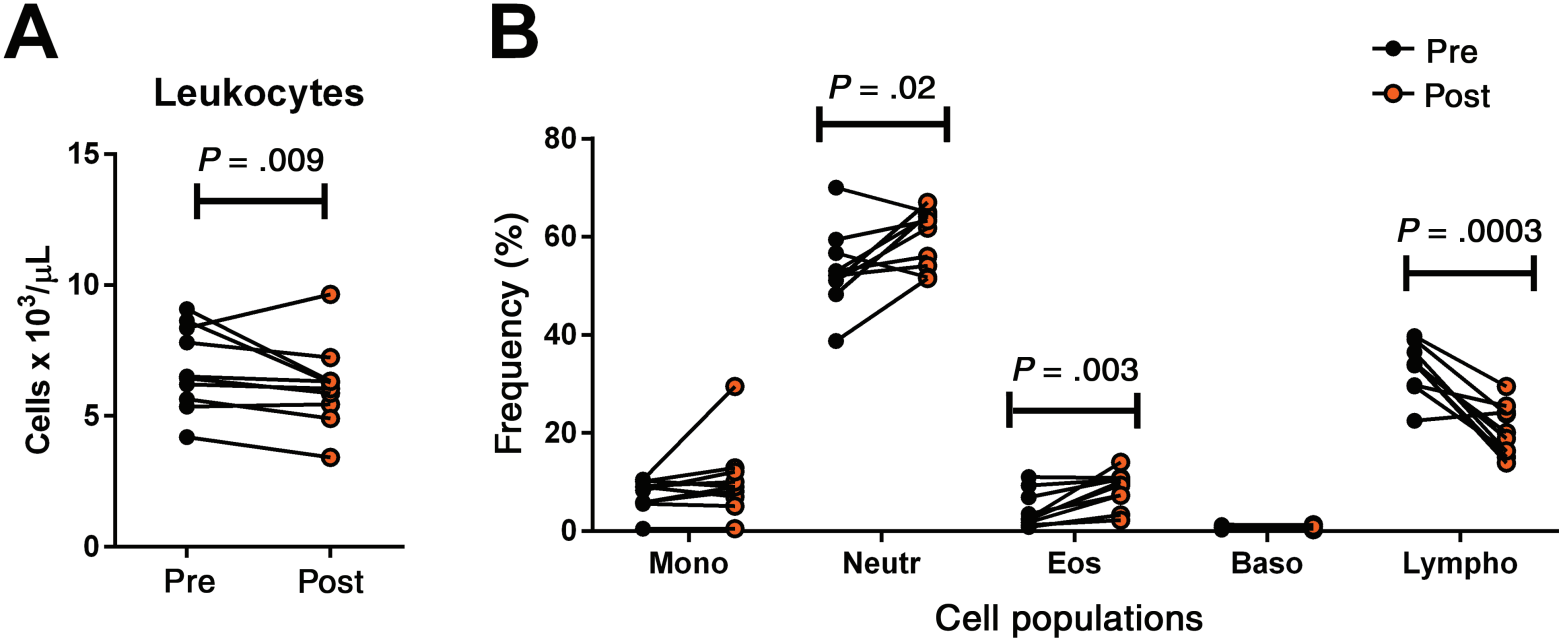
Changes in blood cell counts after meglumine antimoniate treatment. Total leukocyte cell counts (*A*) and the relative frequencies of different cell populations (*B*) were obtained from blood tests performed before and at the end of treatment of each patient (n = 10). Shown are paired data from each patient, denoted as relative frequencies of monocytes (Mono), neutrophils (Neutro), eosinophils (Eos), basophils (Baso), and lymphocytes (Lympho). The statistical difference of means was analyzed and *P* values shown for cell populations with significant differences (*P* < .05).

The kinetics of expression of these clusters showed that modulation of immune gene expression extends beyond EoT (Figure 2B), contrasting with the absence of measurable plasma or intracellular drug concentrations after EoT (Figure 1A). Gene cluster A (neutrophil cluster) had an expression peak at EoT (day 20), while expression of cluster B genes peaked midway through treatment (day 10), with a subsequent decrease to basal levels (based on pretreatment data) observed by EoT. Peak expression of cluster C genes was observed 1 month after EoT (Figure 2B). Consistent with the dynamics of gene expression were the changes in the relative frequency of cell populations in peripheral blood (Figure 3). A significant increase in the relative frequency of granulocytes (neutrophils and eosinophils) was observed at EoT, together with a decrease of total lymphocytes (Figure 3).

### PK/PD Relationships Show Nonlinear Modulation of Immune Response Gene Expression

**Figure 4.**
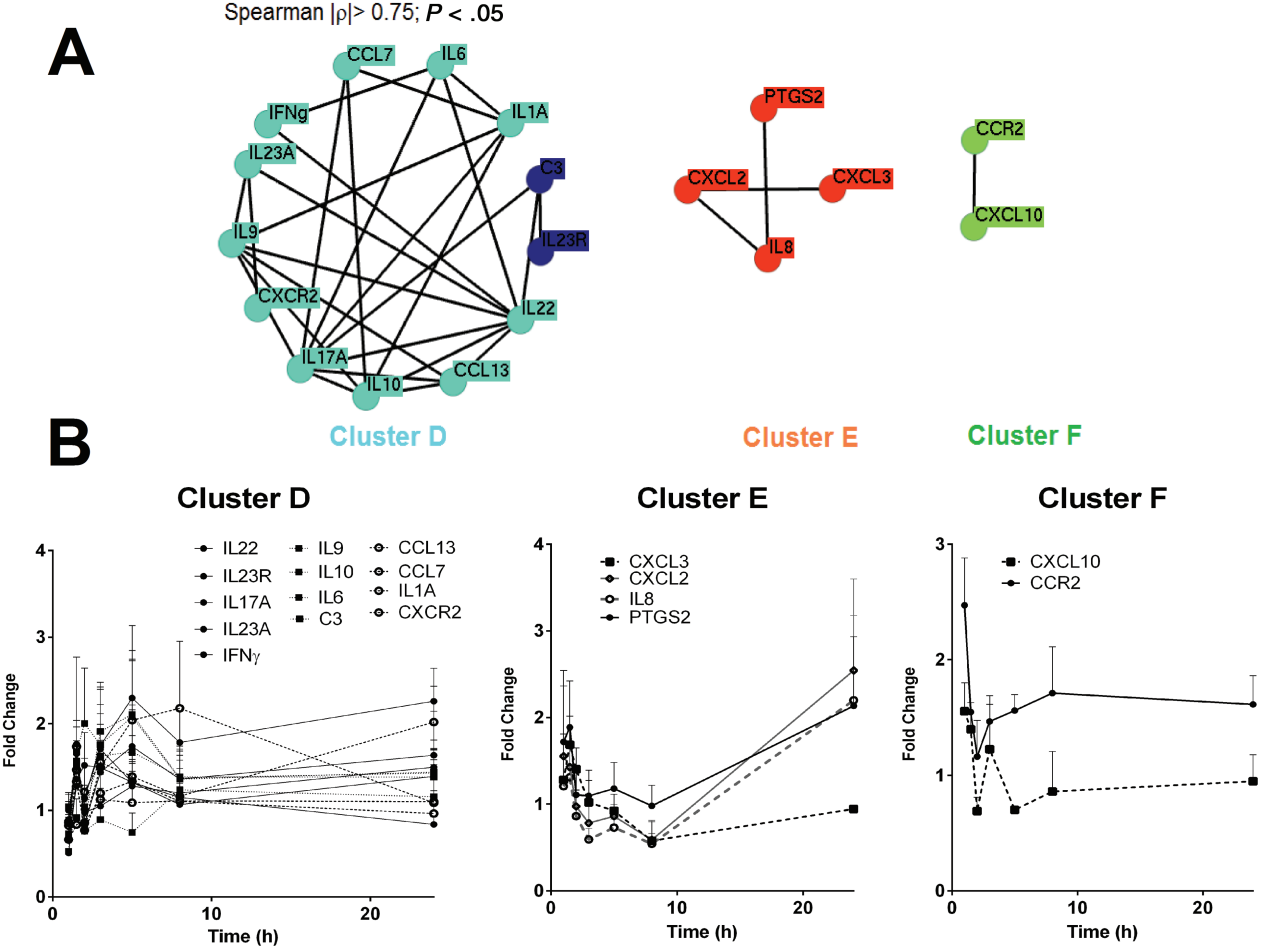
Gene expression networks of peripheral blood mononuclear cells (PBMCs) over the last day of treatment. *A*, Network representation of coregulated or coexpressed genes in PBMCs of cutaneous leishmaniasis patients (n = 4). Represented are genes with significant correlated expression (2^-ΔCt^) over a time course of 24 hours after the final dose of treatment. *B*, Expression profiles of genes within gene clusters. Data are presented as mean values ± standard deviation of the fold-change difference of each gene at each time point over the gene expression of samples collected pretreatment for each patient.

Gene expression was also quantified in PBMCs from 4 CL patients collected during the final day of treatment, at predose, and 1, 1.5, 2, 3, 5, 8, and 24 hours after dosing. Similar to the profiles observed throughout the course of treatment, 3 clusters were generated: a T_h_1/T_h_17 cluster (cluster D) composed of 13 genes: IL1A, CCL13, CCL7, IL6, IFNγ, IL10, IL9, CXCR2, C3, IL17A, IL22, IL23A, and IL23R; the neutrophil cluster containing PTGS2, CXCL3, CXCL2, and CXCL8 (IL8) (cluster E); and cluster F composed of 2 genes, CCR2 and CXCL10 (Figure 4A).

For all genes, a rapid first wave of up- or downregulation was evidenced between 1 and 3.5 hours after dosing (Figure 4B–D), corresponding to plasma and intracellular T_max_ (Figure 1B, Table 2). Overall, cluster E and F genes were downregulated over the first 8 hours after dosing in contrast with cluster D genes, which were overall upregulated. By 24 hours, the level of expression of cluster D genes returned to basal levels, while the levels of expression of cluster E genes slightly increased and the levels of expression of cluster F genes decreased compared with predose expression levels.

A representative gene of each cluster containing 3 or more genes (clusters A/E-CXCL2, B-CCL2, and D-IL22) was selected to explore the relationships between PK parameters and the dynamics of immune gene expression (Table 3). In terms of the short-term effects (within 24 hours), maximum induction of gene expression (E_max,0−24h_) varied among the 3 representative genes: IL22 expression peaked at 1.5 hours after dosing, coinciding with plasma T_max_ (Table 2), while CCL2 peaked at 8 hours and CXCL2 at 24 hours, the latter being the time at which plasma trough concentrations were reached (Figure 1B). Consistently, CXCL2 E_max,d0−90_ occurred at EoT, while E_max,d0−90_ for both CCL2 and IL22 was achieved by day 10.

**Table 3.** Immunological Pharmacodynamic Parameters

Gene Expression Parameter	Cluster A/E *CXCL2*	Cluster B *CCL2*	Cluster D *IL22*
E_max 0−24h_ (FC^a^)	2.54	2.21	1.76
T_max 0−24h_ (hours)	24	8	1.5
E_max 0−90d_ (FC^a^)	1.82	2.57	1.67
T_max 0−90d_ (days)	20	10	10

Abbreviations: E_max_, maximum effect over the time period listed; FC, fold change; T_max_: time of maximum effect over the time period listed.

^a^FC is calculated comparing the expression of each gene at each time point to the expression level pretreatment.

**Figure 5.**
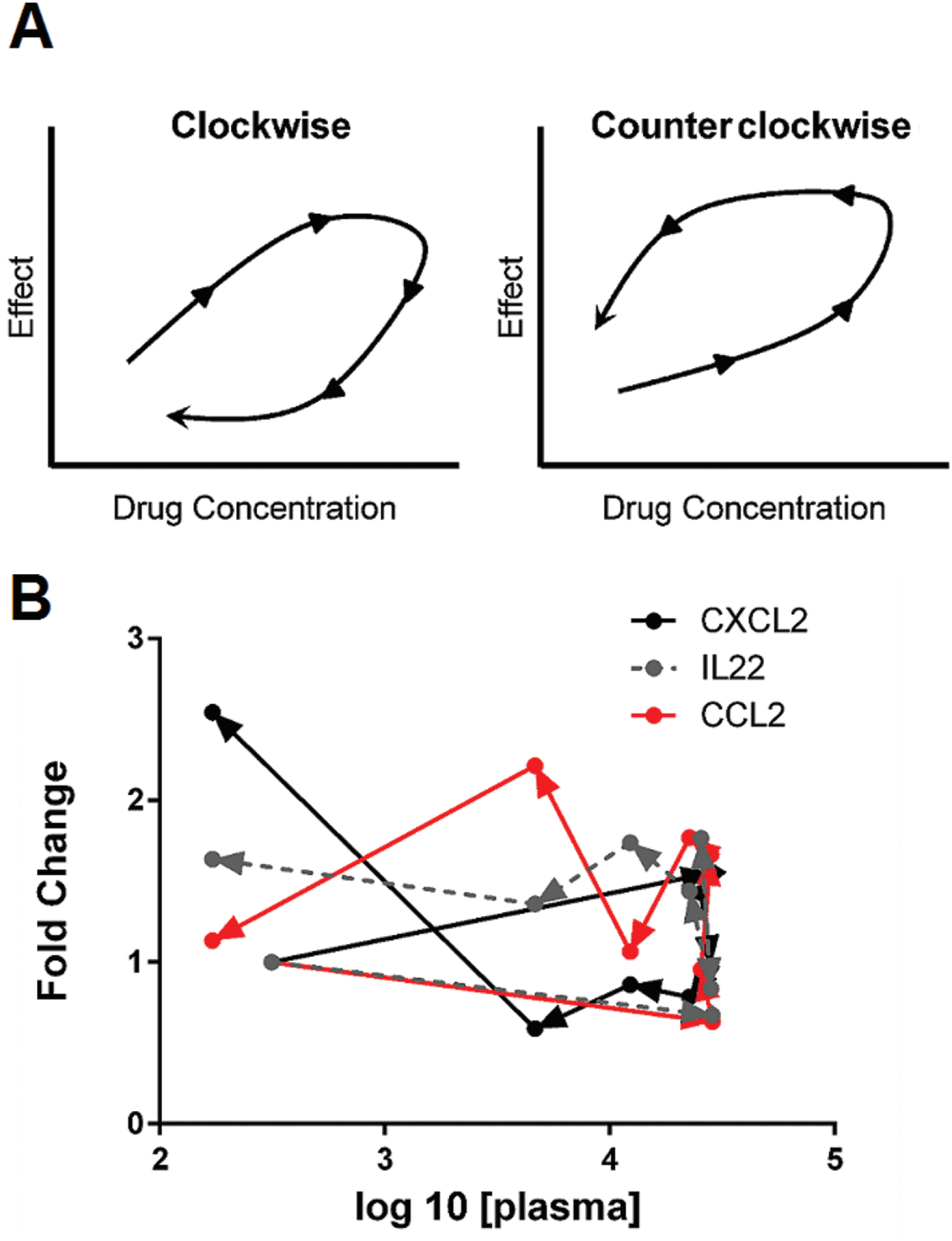
Time-dependent plasma drug concentration-effect curves. *A*, Schematic representation of clockwise and counterclockwise hysteresis loops. Hysteresis loops of plasma antimony concentrations against fold-change expression of genes representative of cluster A/E (CXCL2), cluster B (CCL2), and cluster D (IL22). The directionality of the loops is represented by arrows in each curve. *B*, Clockwise hysteresis shown for CXCL2 (black curve) and counterclockwise hysteresis for CCL2 (red curve) and IL22 (gray dotted curve).

As shown in Figure 5, plasma drug concentration (0–24 hours) vs time-dependent gene expression curves measured at EoT resembled hysteresis loops, which represent nonlinear concentration-effect relationships [26], indicating that similar drug concentrations achieved at different time points of the concentration-time curve (eg, in the ascending and descending segments of the curve) result in different gene expression responses. The directionality of the time-effect curves is represented by clockwise or counterclockwise loops (Figure 5A). Clockwise hysteresis loops are indicative of stronger effects occurring at earlier time points in the concentration-time curve, while counterclockwise hysteresis typically represents stronger effects at later time points [26]. Cluster A/E genes, except for IL1β, as well as cluster B genes, except for CCL2, were represented by clockwise hysteresis (Supplementary Figure 3), as shown by the directionality of the arrows, while cluster D genes (both T_h_1 and T_h_17 genes) were described by counterclockwise loops (Figure 5B and Supplementary Figure 3).

Intracellular Sb concentrations were also plotted against time-dependent gene expression profiles; cluster A genes were linearly and inversely correlated with intracellular drug concentrations (*P* < .02 for all genes, except for PTGS2* P* = .07; Supplementary Figure 4). No relationships could be established for cluster B or D genes. That the hysteresis loops representing the relationship between [Sb_p_] and cluster A gene expression are collapsed when effects are plotted against [Sb_i_] indicates that modulation of genes that mediate neutrophil responses (cluster A) is dependent upon [Sb_i_].

## DISCUSSION

Incomplete understanding of PK/PD relationships of antimicrobials and their relation with the outcome of treatment is a central limitation for optimization of available antimicrobial drugs and regimens [27]. An illustration of this conundrum is therapeutic regimens inferred from plasma PK rather than the effect compartment PK, as a result of restricted access to the affected or target tissues. Furthermore, current applications of PK/PD relationships of antimicrobials aim to limit the emergence of drug-resistant and drug-tolerant microbial pathogens while enhancing elimination of susceptible populations. However, emergence or persistence of drug-resistant/tolerant pathogens is not the sole determinant of treatment failure for infectious diseases, including CL. Orchestrated activation of innate and adaptive mechanisms of microbial kill and tissue repair responses participate in the efficacy of antimicrobials [28–30]. Therefore, parameters such as the minimal inhibitory concentration (MIC), effective concentration (EC_50_), and derived PK/PD indices (eg, AUC/MIC, t > MIC) cannot completely explain complex interactions like those of intracellular pathogens that cause chronic and persistent infections [31–33].

The dynamics of immunological responses during antimicrobial treatment are often considered a consequence of reduction or elimination of the pathogen load, leading to restoration of immune homeostasis; the intensity of the immune response is assumed to depend on the microbial burden [31]. However, as occurs among many other intracellular pathogens, *Leishmania* persists within its human host after clinical cure [34]. Our results demonstrate modulation of systemic immune gene expression signatures immediately after exposure to Sb (as early as 30 minutes after in vivo exposure), indicating that MA, independently of its effect on the parasite load, modifies the expression of immune response genes. We have previously demonstrated partial concordance between some immunological and inflammatory mediators that are modulated at the end of treatment in lesions and PBMCs of CL patients [25]. This finding, together with rapid penetration of Sb into healthy or affected skin following intramuscular dosing [35], suggests that drug-dependent immunomodulation could be occurring rapidly within the active lesion. However, evidence of intracellular Sb accumulation shown in the current study, slower Sb disposition from skin [35], and tissue-specific inflammation that drives dermal pathogenesis, suggest that the dynamics of systemic and local immunomodulation, rather than the type of response, may differ.

Exposure to Sb induces rapid activation of cellular mechanisms of redox control [36–38]. Interestingly, signaling pathways that mediate redox protection can cross talk and influence cellular immune responses [39]. For example, Sb and arsenic strongly induce expression of metallothioneins and glutathione pathway molecules that protect against oxidative stress [40–42]. Metallothioneins participate in intracellular zinc homeostasis, which in turn modulates cell signaling via activity of zinc-binding transcription factors such as NFκB and the enzymatic function of protein tyrosine phosphatases and kinases [43–45]. Functional clustering of genes modulated rapidly after in vivo Sb exposure (clusters A/E, D, and F) is indicative of transcriptional regulation of gene expression. This suggests that a receptor-mediated signaling event is a potential target of Sb drugs in mammalian cells, possibly mediating cellular protection against oxidative stress, as well as immunomodulation.

The unique opportunity to relate plasma and intracellular PK data with immune gene expression profiles in humans has revealed previously unrecognized PK/PD relationships of antimonial drugs. The time-dependent concentration-effect curves for MA and immune gene expression are represented by hysteresis loops. This indicates that identical plasma drug concentrations can result in different gene expression responses, suggesting that the concentration-effect relationships are nonlinear, concordant with nonequilibrium between [Sb_p_] and [Sb_i_] [26]. With the exception of IL1β and CCL2*,* cluster A/E (neutrophil signature) and cluster B genes (Th1 signature) were represented by clockwise hysteresis loops. Clockwise hysteresis is observed when tolerance is developed or feedback regulation is activated because the effect decreases over time with a similar drug concentration [26, 46]. This is concordant with induction of T-cell tolerance and negative feedback regulating highly reactive neutrophils. In contrast, counterclockwise hysteresis loops described all cluster D genes (Th17 and monocyte signatures). Counterclockwise hysteresis is associated with noninstantaneous distribution of the drug to the effect compartment, sensitization or generation of an activating metabolite, suggesting that either Sb-induced monocyte and Th17 responses are susceptible to sensitization, potentially via costimulation with microbial antigens, or that Sb^III^ rather than Sb^V^ leads to modulation of these gene signatures.

Interestingly, correlation analysis of intracellular [Sb] and cluster A/E gene expression data showed an inverse linear correlation, collapsing the hysteresis loops that represented the relationships with [Sb_p_]. The main assumption under the collapse of hysteresis loops is that the measured effect (here, gene expression) depends on drug concentrations in the effect compartment (here, PBMCs) rather than the central compartment [26]. Therefore, our data suggest that intracellular Sb may signal for expression of neutrophil-activating and chemotactic CXCL chemokines as well as for IL1β and PTGS2. Among the central regulators of innate immune signaling are Toll-like receptors (TLRs), and their activation results in expression of proinflammatory chemokines (including cluster A/E chemokines CXCL2, CXCL3, and IL8), cytokines, and importantly IL1β. Although originally described as pattern recognition receptors that (PRR) mediate responses to pathogens, TLRs can also recognize a multitude of molecules, including metals such as nickel, cobalt, and palladium, among others [47, 48], and metal-based nanoparticles [49]. Whether Sb can signal through TLRs or other PRRs remains to be determined.

The PK/PD relationships described by hysteresis loops concur with the following three features of the observed PK/PD relationships of MA:

(1) the time-lag between [Sb_p_] and [Sb_i_] indicates nonequilibrium between the central and the effect compartment [Sb]. (2) Sb^V^ is reduced to Sb^III^ for direct antileishmanial effect, and this can occur in mammalian as well as *Leishmania* cells [50–52]. However, the high reactivity of free Sb^III^ needs to be rapidly controlled to minimize cell damage, which typically occurs through Sb^III^ -glutathione complexation coupled to other mechanisms of metal-induced detoxification [53]. Thus, Sb^III^ bioavailability may differ at different time points after dosing, resulting in differential effects over immune gene expression. (3) The immune response is susceptible to sensitization and tolerance; cross talk of signaling pathways and regulatory loops determines the magnitude and directionality of cellular responses. Microbial, cytokine, and other stimuli and stressors can potentiate or dampen these responses through modulation of signaling cascades at the receptor, signal transducer, or transcriptional levels.

A second dimension of gene regulation was observed in our study, represented by a long-term synchronized modulation of systemic immune networks throughout the course of treatment. Our results show a first peak of responses measured midway through treatment, suggesting recruitment of proinflammatory cells (monocytes and Th1 cells) to the periphery, followed by recruitment of activated granulocytes at EoT. We interpret these dynamics as the restoration of immune homeostasis, reorienting inflammatory cell trafficking from the inflamed tissue into circulation and limiting the contribution of local inflammation to the cutaneous immunopathology. These more regulated waves of gene expression likely represent the combined outcome of drug-dependent and parasite load–dependent immunomodulation.

Modulation of systemic immune gene expression profiles throughout the course of antileishmanial treatment, together with evidence of intracellular Sb accumulation despite lower [Sb_i_] compared with [Sb_p_], provides new bases for optimization of antimonial drugs through improved drug delivery systems (DDSs). To reduce toxicity and length of antileishmanial drug regimens, DDSs have been empirically selected assuming that their main benefit arises from enhanced drug concentration within infected macrophages at different anatomical sites [54]. However, the consistently poor efficacy of encapsulated antileishmanials for treatment of CL raises doubts about their clinical applicability [54]. The data presented here provide a new frontier of inquiry for the efficacy of drugs for CL, introducing immune gene expression profiles as PD parameters in addition to the classic measurement of direct antileishmanial effects. Our results provide a reference for determining target intracellular drug concentrations, target cell populations (beyond macrophages), and target gene signatures to identify formulations that potentiate the hysteresis loops, favoring an immune profile of healing as well as reduction of the parasite burden.

## Supplementary Data

Supplementary materials are available at *Clinical Infectious Diseases* online. Consisting of data provided by the authors to benefit the reader, the posted materials are not copyedited and are the sole responsibility of the authors, so questions or comments should be addressed to the corresponding author.

ciaa1206_suppl_Supplementary_Material

## Notes

***Author contributions.*** M. A. G. study design, analysis and interpretation of results, and writing of manuscript versions. A. N. development of experimental approaches, analysis and interpretation of results, and revision of manuscript versions. M. P. clinical management of the study protocol and patient enrollment, analysis and interpretation of results, and revision of manuscript versions. L. G.-P. development of experimental approaches, analysis and interpretation of results, and revision of manuscript versions. A. C. clinical management of the study protocol and patient enrollment, analysis and interpretation of results, and revision of manuscript versions. N. A. study design, data analysis and interpretation of results, and revision of manuscript versions. N. G. S. study design, data analysis and interpretation of results, and revision of manuscript versions.

***Acknowledgments.*** The authors give special thanks to all study participants for their invaluable contributions. We acknowledge the invaluable and dedicated support of the clinical group of CIDEIM in Cali, Colombia, and Tumaco, Colombia, in the implementation and conduct of the complex research protocol. We also thank Dr David Esteban Rebellón for his support with the case report files and other clinical data. M. A. G. specially thanks Dr Antonio Guerra Guerra for illuminating and enabling inspiration.

***Financial support.*** This research received support from a Wellcome Trust award (107595/Z/15/Z) and from the National Institute of Allergy and Infectious Diseases (NIAID) of the National Institutes of Health (NIH; under award U19AI129910).

***Potential conflicts of interest.*** N. G. S. reports funding from the NIH NIAID and the Fogarty International Center, a Special Programme for Research and Training in Tropical Diseases (TDR) Special Program hosted by World Health Organization, and the Swiss National Fund unrelated to the submitted work. All other authors report no potential conflicts. All authors have submitted the ICMJE Form for Disclosure of Potential Conflicts of Interest. Conflicts that the editors consider relevant to the content of the manuscript have been disclosed.
