# Supplementary material for: Immuno-pharmacokinetics of Meglumine Antimoniate in Patients With Cutaneous Leishmaniasis Caused by Leishmania (Viannia)
Source: Clin Infect Dis. 2020 Aug 20;72(10):e484–92. doi: 10.1093/cid/ciaa1206 (PMC8130027; doi:10.1093/cid/ciaa1206)
Supplement: ciaa1206_suppl_Supplementary_Material [file ciaa1206_suppl_supplementary_material.docx]

**Immuno-pharmacokinetics of meglumine antimoniate in patients with cutaneous leishmaniasis caused by *Leishmania Viannia*.**

María Adelaida Gómez^1,2*^, Adriana Navas^1,2^, Miguel Prieto^1^, Lina Giraldo-Parra^1,2^, Alexandra Cossio^1,2^, Neal Alexander^1^, Nancy Saravia^1,2^

**Supplementary Material**

**Supplemental Methods**

**Procedures and samples.**  CL patients: prior to enrollment, each potential participant was examined and baseline evaluations were performed including blood cell counts and assessment of cardiac, pancreatic, liver and renal function; blood tests were repeated at the end of treatment (EoT) to monitor adverse events. Patients were treated with a standard course of parenteral meglumine antimoniate (20 mg Sb/kg every 24h for 20 days) [[18](#_ENREF_18)]. Clinical and laboratory follow-up included visits at the first day of treatment, at days 10 and 20 (EoT) during treatment, and at weeks 8 and 13 (± 2 weeks) after initiation of treatment, the latter time at which clinical outcome was determined. Cure was defined as complete re-epithelialization, absence of inflammatory signs for all CL lesions, and absence of new leishmaniasis lesions [[19](#_ENREF_19)].

Blood specimens were obtained from an arm vein and anticoagulated with EDTA for measurement of antimony concentrations in plasma, or with heparin for gene expression analyses. A control sample was obtained immediately prior to initiation of treatment, and additional samples were collected 1h post-dose on days 1, 10 and 20, and on weeks 8 and 13 after initiation of treatment. Patients remained at CIDEIM outpatient clinics in Cali on day 20, when the PK sampling was conducted: sequential samples were obtained at 0, 0.5, 1.0, 1.5, 2, 3, 5, 8, 12 and 24 h after the final dose (Flow diagram in Supplemental Figure 1). Plasma and peripheral blood mononuclear cells (PBMCs) were isolated from these samples.

Blood samples from three healthy volunteers were used for parallel isolation of monocytes and PBMCs for *ex vivo* assays of intracellular Sb accumulation. All samples were immediately stored at –80°C.

**Isolation and processing of PBMCs.** PBMCs were isolated by centrifugation over a Ficoll-Hypaque 1077 gradient (Sigma), quantified and their viability assessed using trypan blue. Cells were cryopreserved as dry pellets at -80°C. For gene expression analysis, and measurement of intracellular drug concentrations, frozen dry pellets were immediately resuspended in Trizol or 25% (w/w) tetramethyl ammonium hydroxide (TMAH), respectively.

**ICP-MS run:** Prior to initiating the run, high and low system suitability tests were performed using the upper and lower limit of quantitation (LOQ). Each analytical run included validation, quality control and calibration curves using ICP-MS grade Sb, and injected at the beginning and end of the run. Each sample included indium as an internal standard. Quality control samples were included in two sets of low, medium, and high concentrations interspersed throughout the analytical run with study samples.

**Intracellular drug concentrations:** Sb concentrations in PBMCs were calculated by determining the total Sb content of the pellet (digest concentration × digest volume), followed by total Sb per cell (digest concentration × digest volume /total number of PBMCs), and dividing by the total cell volume (where 283 fL is the average volume of a PBMC [[20](#_ENREF_20)]).

**RNA extraction**: Total RNA from PBMCs was extracted using Trizol reagent (Invitrogen, USA) followed by RNA cleanup with RNeasy Mini Kit Columns (Qiagen, USA). The quantity and quality of the extracted RNA was evaluated in a Nanodrop ND-1000 spectrophotometer, and RNA integrity confirmed by agarose gel electrophoresis. cDNA was synthesized using the RT first strand synthesis kit (Qiagen). RT-qPCR reactions were run on a CFX96 Real Time System (Bio-Rad).


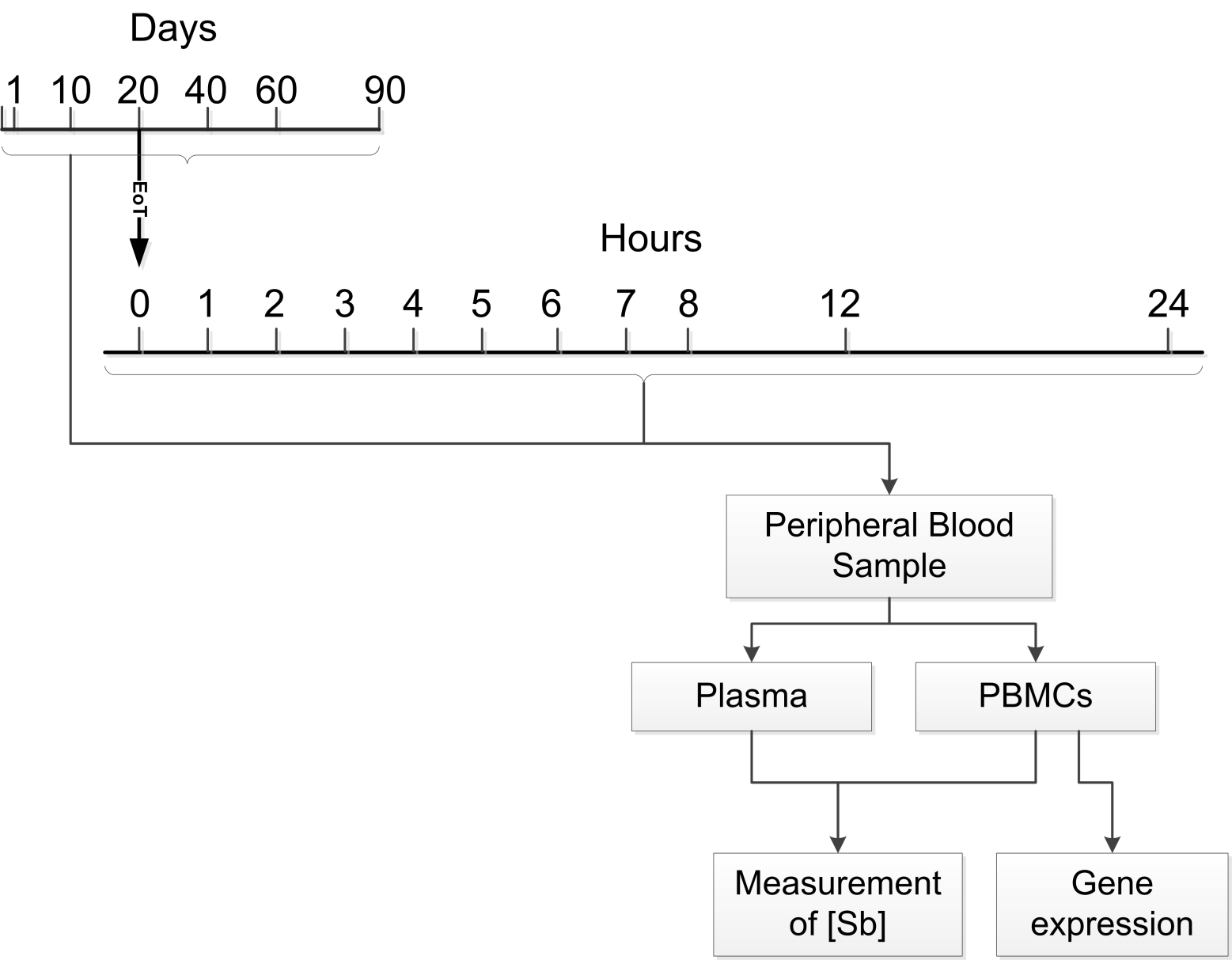


**Figure S1**. Flow diagram representing the blood sampling scheme for CL patients and the laboratory procedures for each sample.


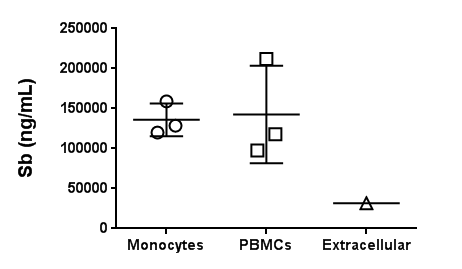


**Figure S2. Intracellular Sb in PBMCs and monocytes.** PBMCs and monocytes where isolated from peripheral blood samples from three healthy donors. Ten million monocytes (open circles) or PBMCs (open squares) were incubated for 1 h in 2mL RPM1 containing 10% FBS and 32,000 ng/mL Sb (as meglumine antimoniate), and then washed once with PBS Cell pellets were suspended in 25% TMAH. Intracellular Sb concentrations were measured by ICP-MS. Extracellular Sb concentration (open triangles) was measured in culture supernatant of exposed cells, to corroborate the drug concentration during exposure.


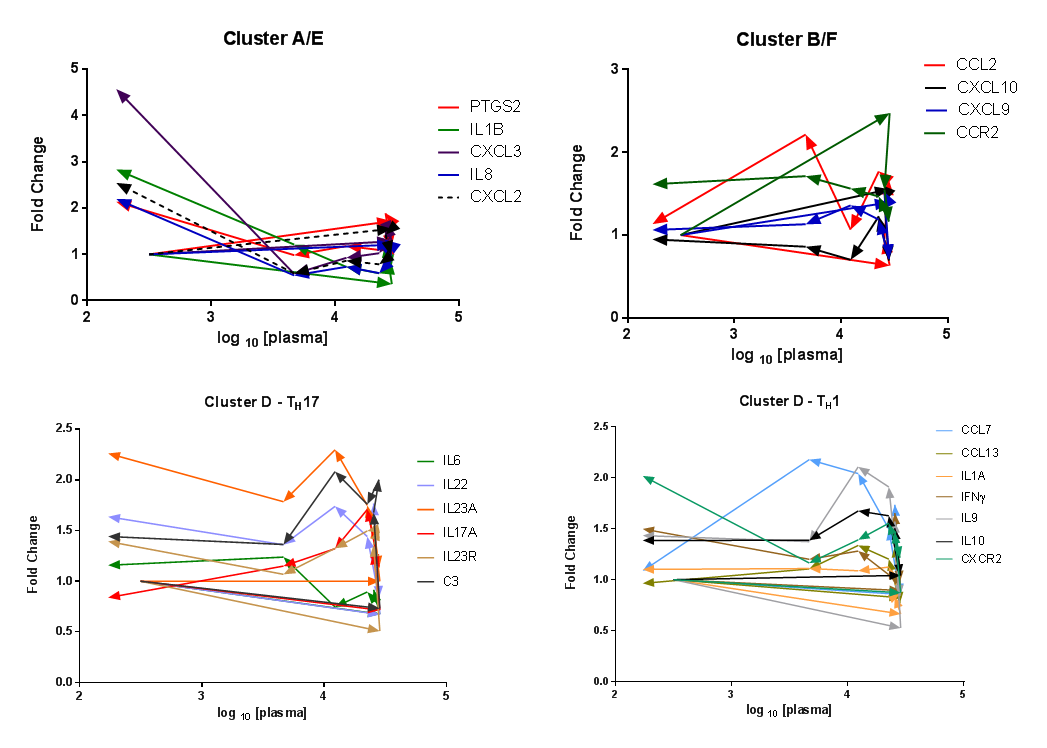


**Figure S3. Hysteresis loops representing concentration-effect relationships for all genes within gene clusters.** Hysteresis loops of plasma Sb concentrations against fold change expression of all genes constituting each gene cluster. Represented are genes within clusters A/E, B/F and D. Cluster D genes have been subdivided within two categories for simplicity of graph interpretation (cluster D – Th17 related genes, and Cluster D – Th1 related genes). The directionality of the loops is represented by arrows in each curve.


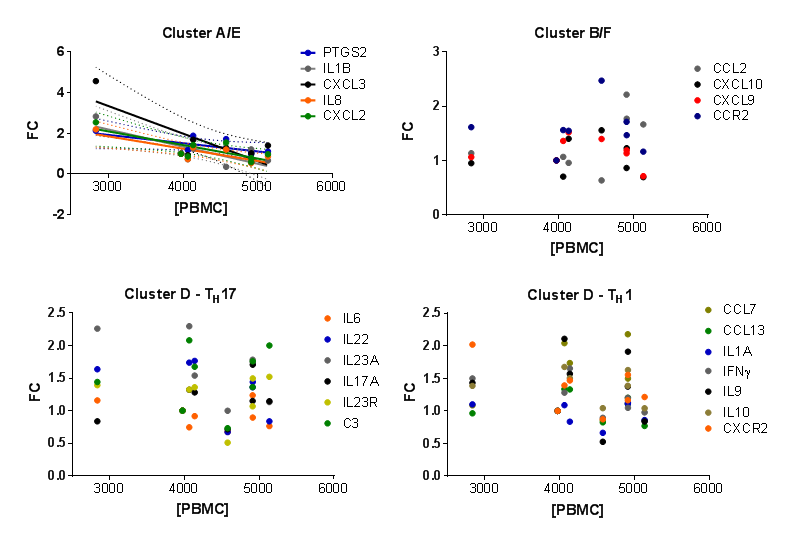


**Figure S4. Relationships between gene expression and intracellular drug concentrations.** Correlation plots of [Sb_i_] against fold change expression of all genes constituting each gene cluster. Represented are genes within clusters A/E, B/F and D. Cluster D genes have been subdivided within two categories for simplicity of graph interpretation (cluster D – Th17 related genes, and Cluster D – Th1 related genes). Cluster A/E includes linear regression estimates for each gene (continuous lines) and 95% Confidence intervals (dotted lines).

**Table S1. Custom-made PCR Array gene content and gene product functions**

| **Functional cluster** | **Gene symbol** |
| --- | --- |
| **Monocyte/macrophage activation and recruitment** | CCL2, CCL7, CCL13, CD14, CSF1, TNFα, TLR7, CCR2, IL1A, IL1β, IL18 |
| **Neutrophil activation and recruitment** | CXCL2, CXCL3, CXCL8, CXCR2, C3 |
| **Th1 cell activation and recruitment** | CXCL9, CXCL10, IFNγ |
| **Th17 activation and recruitment** | IL6, IL22, IL23A, IL17A, IL23R |
| **Anti-inflammatory cytokines and enzymes** | IL10, IL9, PTGS2 |
